# Complications during Adolescence Predict Mortality in Young Adults with Childhood Onset Type 1 Diabetes

**DOI:** 10.1155/2024/8194756

**Published:** 2024-04-12

**Authors:** Myra S. Poon, Albert K. F. Chan, Janine M. Cusumano, Maria E. Craig, Kim C. Donaghue

**Affiliations:** ^1^Institute of Endocrinology and Diabetes, The Children's Hospital at Westmead, Sydney, New South Wales, Australia; ^2^Discipline of Paediatrics and Child Health, University of Sydney, Sydney, New South Wales, Australia; ^3^Discipline of Paediatrics and Child Health, School of Clinical Medicine, UNSW Medicine, Sydney, New South Wales, Australia

## Abstract

**Objective:**

Microvascular complications increase the risk of cardiovascular disease and premature death in adults with type 1 diabetes. We examined the association between microvascular complications during adolescence, including cardiac autonomic nerve dysfunction and subsequent mortality. *Research Design and Methods*. We undertook data linkage with the Australian National Death Index in a cohort of 409 adolescents (diagnosed between 1973 and 1993), 48% male, median age at final complications assessment 17.4 years (interquartile range: 16.0–18.9), followed longitudinally for median 22.3 years (21.0–23.4) from diagnosis. Generalized estimating equations (GEE) were used to examine associations between mortality and adolescent complications. Mortality risk was calculated as standardized mortality ratio (SMR).

**Results:**

At final adolescent visit, 20% had CAN abnormality, 30% abnormal pupillary response, 20% albuminuria, 40% early elevation of albumin excretion rate (AER) and 45% retinopathy. Data linkage 8–13 years later showed 14 were deceased (3% of cohort), 57% male, median age 28.3 years (24.8–32.9). Acute or chronic diabetes complications accounted for 25% of deaths. In multivariable GEE, elevated AER (OR 4.54, 1.23–16.80, *p*=0.030), pupillary abnormality (OR 4.27, 1.20–15.22, *p*=0.023), systolic blood pressure SDS (OR 2.17, 1.26–3.74, *p*=0.005) and CAN (OR 4.65, 1.03–21.0, *p*=0.045) predicted mortality. HbA1c was not significant. SMR was 2.5 (1.4–4.2) and was higher in females (SMR 3.5, 1.3–7.8) but not in males (SMR 2.1, 0.9–4.0).

**Conclusion:**

Mortality in young adults with type 1 diabetes is predicted by subclinical markers of autonomic neuropathy and elevated AER during adolescence, but not glycemia. Mortality was over twice that of the background population in females but not in males.

## 1. Introduction

Type 1 diabetes is associated with increased mortality compared with the general population, despite improvements in diabetes management and strategies for optimizing glycemic targets over recent decades. Recent studies indicate that type 1 diabetes reduces life expectancy by up to 12.9 years [1, 2] and standardized mortality ratio (SMR) ranges from 2.0 to 7.55, depending on the region studied [1, 3, 4]. Although mortality rates have declined across some countries in recent decades, SMR remains high when compared with the population without type 1 diabetes [5–7].

While there are multiple causes of premature death in adults with type 1 diabetes, excess mortality may be attributable to the autonomic nerve dysfunction. This association was initially reported by Ewing et al. [8], who demonstrated mortality rates of 53% in symptomatic individuals with abnormal autonomic tests compared with 15% in those with normal tests [8]. A meta-analysis of mortality and cardiac autonomic neuropathy [5] demonstrated a relative risk of 3.76 for all-cause mortality in the subgroup analysis of people with type 1 diabetes [9].

Symptoms of autonomic dysfunction may be subtle and remain unrecognized until late in the disease course. However, subclinical autonomic neuropathy may be detected even in young people, using relatively simple and noninvasive tests. In a meta-analysis, the pooled prevalence of cardiac autonomic dysfunction and abnormal pupillometry was 28% and 42%, respectively [10].

The majority of previous studies of autonomic dysfunction and mortality have only included adults (mean ages: 33–65 years) with relatively a short follow-up [11]. In a 12-year follow-up study, we previously found that adolescent autonomic dysfunction predicted subsequent microvascular complications of those reassessed during young adulthood [12]. In the present study, we expanded the cohort and linked it to the National Death Index to examine whether subclinical autonomic dysfunction during adolescence predicts mortality in young people after type 1 diabetes duration of more than 20 years. We also investigated whether other microvascular complications and glycemia predict mortality.

## 2. Methods

### 2.1. Study Population

This longitudinal cohort of 409 young people underwent baseline diabetes complications assessment between 1990 and 1995 (diagnosed between 1973 and 1993, 48% male, median age: 14.4 years, duration: 6.2 years), as per standard practice according to contemporary guidelines (International Society of Paediatric and Adolescent Diabetes) [13]. This cohort comprised consecutive adolescents assessed for complications during this period. Earlier follow-up of part of this cohort was previously reported for adolescents undergoing complications assessment between 1990 and 1993 [12].

### 2.2. Diabetes Complications Assessment

Participants were assessed using a structured interview and clinical examination including auxological measurements (height and weight) and resting blood pressure (BP). Systolic and diastolic BP measurements were converted to standard deviation scores (SDS) adjusted for age and sex [14]. SDS of body mass index (BMI) were based on age- and sex-related reference standards [15].

Cardiac autonomic nerve function during adolescence was assessed by three tests of heart rate variation (maximum–minimum heart rate during deep breathing (DBT) – abnormal < 22,) and lying to standing heart rate change (30 : 15 ratio) – abnormal < 1.08 (LSHR). Change in BP from lying to standing (PBPT) was also assessed with abnormal defined as systolic BP fall of >13 mm Hg. CAN ranges used were derived from 122 nondiabetic controls [16] and abnormal was defined as less than the fifth percentile for each test. Participants were classified as having subclinical CAN if an abnormality was detected on at least one test.

Pupillary autonomic function during adolescence was assessed using an infrared pupillometer (Pupilscan, Fairvill Medical Optics) as previously described [17]. Parameters included were pupil diameter at rest and 3 s after a light stimulus was delivered, reflex pupillary amplitude, and maximum constriction velocity. Reference ranges were derived from 122 nondiabetic control subjects. Abnormal pupillary tests were defined as less than the fifth percentile of the reference range. Resting pupil diameter < 4.77 mm was also assessed as a variable, with abnormal defined as within the lowest quartile as this predicted albuminuria and retinopathy in a subgroup examined previously [18].

Retinal screening was performed using photographs taken with a Topcon Fundus camera (TRC 50-VT, Tokyo Optical Co., Tokyo) after dilatating the pupils with 1% cyclopentolate and 2.5% phenylephrine. Nonsimultaneous photographic pairs were taken of seven standardized fields in each eye and then viewed using a Donaldson Stereoviewer. This provided a three-dimensional representation of the fundus and enabled microaneurysms to be more easily distinguished from hemorrhages and artifacts. The photographs were graded by the same ophthalmologist, who blinded to the participant's complications status, according to the Early Treatment Diabetic Retinopathy Study (ETDRS) adaptation of the modified Airlie House classification of diabetic retinopathy. Retinopathy was defined as the presence of at least one microaneurysm or hemorrhage (grade 21 or higher), as previously described [19].

Peripheral nerve function was assessed by hot and cold thermal threshold testing of the left foot using the TSA-II Neurosensory Analyzer (Medoc Ltd.). Vibration threshold at the left medial malleolus and left great toe was measured with the VAS-3000 Vibratory Sensory Analyzer (Medoc Ltd.). Peripheral nerve abnormalities were defined as >95% of the normal range in a nondiabetic adolescent control group, as previously described.

### 2.3. Biochemical Measurements

Albumin was measured using polyclonal radioimmunoassay (Pharmacia RIA, Beckman Coulter, Australia) prior to 2000, nephelometric assay using an IMMAGE analyser (IMMAGE = (0.8734 × radio-immunoassay value)−0.501; *r* = 0.99) from 2000 to 2003 and competitive chemiluminescence immunoassay using the IMMULITE analyser (Diagnostic Products, Los Angeles, CA) thereafter. Creatinine was measured by Jaffe reaction, Dimension ARX (Dade Behring, Newark, DE). Early elevation of albumin excretion rate (AER) was defined as a mean excretion rate >7.5 *μ*g/min. Albuminuria was defined as AER > 20 *μ*g/min or spot albumin–creatinine ratio (ACR) ≥ 3.5 mg/mmol for males or ACR ≥ 4.0 mg/mmol for females in two out of three timed consecutive overnight urine samples. HbA1c was measured using high-performance liquid chromatography (Diamat Bio-Rad analyser, Bio-Rad, Herculus, CA; nondiabetic range 4%–6%).

### 2.4. Estimation of Mortality Rates

Mortality data were obtained by linkage with the National Death Index (NDI) [20] (maintained by the Australian Institute of Health and Welfare) in February 2014, including all deaths from date of diabetes onset until the linkage date. Records at that time were current until mid-December 2013. Secondary ascertainment of deaths was performed by crosschecking with the records held by Diabetes Australia. This is a consumer organization that assists with the administration of the National Diabetes Services Scheme to provide access to subsidized insulin and equipment.

Cause of death was available from the NDI for the majority of individuals and coded according to the International Classification of Diseases (ICD)-9 for deaths prior to the end of 1996 and by ICD-10 thereafter. National standardized death rates from the Australian Bureau of Statistics for the relevant age groups were used to calculate the number of expected deaths in the cohort.

### 2.5. Statistical Analysis

Descriptive statistics is reported as frequency (%) for categorical variables, mean ± standard deviation (SD) for normally distributed variables, and median (interquartile range, IQR) for continuous variables. Differences in proportions were calculated using chi-squared tests and differences in median were calculated using the Mann–Whitney *U*-test.

Results of all complications assessments were included in the analysis. Generaize estimating equations (GEE) were used to examine the association between the outcome (mortality at data linkage) and explanatory variables (measured at all participant visits) including cardiac autonomic nerve abnormality, pupillary abnormality, retinopathy, peripheral neuropathy, elevated AER, albuminuria, severe hypoglycemic episodes, HbA1c, BMI-SDS, systolic and diastolic BP-SDS, age at diagnosis, age at visit and sex. Results of univariate and multivariable models are reported as odds ratio (OR) and 95% CIs.

Standardized mortality ratio (SMR) was calculated as observed deaths/expected deaths for age- and sex-matched population over the time period studied and 95% confidence intervals were calculated using a Poisson distribution.

Analyses were performed using SPSS v25 (IBM statistics, Armonk, NY).

## 3. Results

Data totalling 9,025 person-years of follow-up were analyzed. Median diabetes duration at time of data linkage was 22.3 years (IQR: 21.0–23.4) at February 2014 and maximum number of visits was 10.

Fourteen individuals were deceased (3% of the cohort, 57% male, median age at death 28.3 years (24.8–32.9), age range: 19.1–38.1 years). Median duration of diabetes at death was 19.6 years (13.5–26.0) and median lifetime HbA1c was 8.2% (7.2–9.6) (66 mmol/mmol (55–81)).

An acute complication of diabetes was the primary cause of death for 4/14 (25%). One of these deaths was associated with severe hypoglycemia and three were classified as due to diabetes with or without ketoacidosis (DKA). Mean HbA1c was 10% (86 mmol/mmol) for the latter three and all had elevated AER. Mean age at death was 26.3 ±8.7 years and duration 19.1± 10.7 years.

Chronic kidney disease was the primary cause of death for one person (6%) who had attended two assessments. Urine AER was unfortunately not available at either visit for this individual. Four died in road traffic accidents. Further details, such as whether the deceased was the driver or passenger, were not available; however, all were aged over 18 years at the time of death. Malignancy was the cause of death in three individuals: chronic myeloid leukemia and skin and connective tissue malignancies with secondary malignancies in the lung. Intracranial hemorrhage and opioid dependence each accounted for one death.

We compared HbA1c between those whose cause of death was diabetes-related (diabetes with or without diabetic ketoacidosis, hypoglycemia, chronic kidney disease, *n* = 5) and those whose cause of death was not related to diabetes and found no significant difference (mean HbA1c 9.6%± 2.0% vs. 8.9% ± 1.9%, *p*=0.24).

Characteristics at the final visit of the whole group, alive and deceased, are presented in Table 1. At the final complications assessment visit, the proportion of individuals with a CAN abnormality was higher in the deceased group compared with the group still alive at follow-up (46% vs. 19%, *p*=0.02). There was no significant difference between the two groups in other parameters at the final visit.

In the deceased group, retinopathy was present in 27%, elevated AER in 50%, and peripheral neuropathy in 31%. None of the deceased individuals had albuminuria at the final visit. CAN abnormality was detected in 46% and pupillary abnormality in 38%.

In univariate analysis, mortality was significantly predicted by cardiac autonomic neuropathy, systolic blood pressure-SDS, elevated AER and peripheral nerve abnormality. There was some evidence for pupillary abnormality, but it did not reach statistical significance. Mortality was not predicted by median HbA1c during adolescence, retinopathy, BMI-SDS, diastolic blood pressure-SDS, albuminuria, nor history of severe hypoglycemia (Table 2). In addition, sex (OR 1.00, 0.99–1.01, *p*=0.28), age at diagnosis (OR 0.95, 0.80–1.12), *p*=0.52) and age at visit (OR 1.00, 0.99–1.01, *p*=0.28) were also examined and did not predict mortality.

The univariate predictors which were found to be statistically significant in the multivariable model are shown in Table 2. In multivariable analysis, elevated AER, pupillary abnormality, systolic blood pressure SDS and cardiac autonomic neuropathy were significant predictors of mortality. There were no significant interactions between variables.

All-cause mortality was 1.19/1,000 person-years of diabetes. The overall SMR was 2.5 (95% CI: 1.4–4.2). When stratified by gender, only the SMR for females was significantly increased (3.5, 95% CI: 1.3–7.6). The SMR for males was 2.1 (95% CI: 0.9–4.0).

## 4. Conclusions

Our prospective cohort study of young adults with type 1 diabetes is the first to demonstrate that subclinical autonomic neuropathy, early elevation of AER, and higher systolic blood pressure during adolescence predict mortality after more than two decades.

Our findings are consistent with the EURODIAB study which demonstrated that macroalbuminuria, peripheral, and autonomic neuropathy were the most important risk markers for mortality, with the latter associated with three times the risk of death. However, this population was significantly older at baseline [21]. In our cohort, we have demonstrated that even young people with early signs of microvascular complications and subclinical autonomic neuropathy are at increased mortality risk.

The association between diabetic nephropathy, cardiovascular disease, and mortality has been extensively documented [20]. Furthermore, Orchard et al. [11] described a cohort of young adults with cardiac autonomic neuropathy in which increased mortality was related to the presence of nephropathy. This cohort was diagnosed in an earlier time period (1950–1980) and only one test of cardiac autonomic neuropathy was performed [11]. Another small study demonstrated an association between autonomic dysfunction, characterized by reduced 24-hr electrocardiogram RR interval variability, microalbuminuria, and macroalbuminuria, supporting the relationship between these two complications [22]. These findings represent a reduction in parasympathetic activity and a possible relative increase in sympathetic activity, a pattern that is associated with an increased risk of sudden death in ischemic heart disease [23].

Although early elevation of AER predicted mortality in our cohort, the presence of albuminuria did not. However, the rate of albuminuria was only 8%, and therefore, the small number of individuals with this complication may have contributed to the lack of association.

Autonomic dysfunction may be associated with a prolonged QTc [24] and increased mortality [25]. Autonomic dysfunction and hypoglycemia have been proposed as a mechanism underlying the “dead in bed” syndrome [26]. We were unable to determine whether the two deaths attributed to hypoglycemia and acute diabetes complications could have been classified as “dead in bed.” However, this devastating complication remains a concern in young people with type 1 diabetes.

We found that HbA1c during adolescence did not predict mortality, in contrast to findings from a West Australian population cohort [27]. In adults, higher mortality rates are associated with increasing HbA1c [28, 29]. We have demonstrated that even early evidence of cardiac autonomic neuropathy and early elevation of AER during adolescence can predict mortality. Therefore, in adolescence, robust measures, such as microvascular complications, appear to be more informative of mortality risk than HbA1c.

Acute and chronic complications of diabetes accounted for approximately one-third of deaths, consistent with reports of individuals of similar age [1, 2] and slightly lower than a Norwegian cohort [30]. In previously published studies, many deaths have been attributed to CAN and renal disease in older age groups [31], whereas acute metabolic complications accounted for a greater proportion of deaths in those aged <40 years [32]. Some of the remaining deaths may have been attributable to diabetes. In the case of individuals who died in road traffic accidents, it was not possible to determine whether the person with diabetes was the driver or passenger and whether the accident could have been related to diabetes.

Diabetes has been associated with an increased rate of unnatural or violent deaths an increased risk of suicide attempts, suicidal ideation, and violent deaths [33–35] as well as alcohol-related deaths [36]. Five deaths (36%) in our cohort could be classified in this group. In a Swedish study, the risk ratio for suicide was 6.7 (95% CI: 4.27–10.50) and 7.57 (95% CI: 2.88–3.68) for accidents when compared with the general population. This group was also overrepresented among the cases of poisoning by unspecified drugs or medication. In the subgroup who died by suicide, 30% were poisonings with insulin or oral hypoglycemic agents [37]. These findings may reflect the increased psychological burden associated with long-standing type 1 diabetes.

Overall SMR was 2.5-fold higher for males and 3.5-fold higher for females. These mortality rates are comparable with population-based Swedish and Norwegian studies [4, 33], and slightly lower than a young adult Norwegian cohort with similar diabetes duration [36]. Similar to a recent meta-analysis, the mortality risk was higher for females than for males [38]. Several studies have demonstrated that life expectancy is reduced by 11–17 years compared with the age-matched populations [1, 2]. A Swedish study has shown improvement in life expectancy for men, but not women, over the period from 2002 to 2011.

Limitations of this study include the possibility of attendance bias among individuals who achieve HbA1c targets. Indeed, in the previous follow up of this cohort, participation was greater in those with lower glycated hemoglobin measurement when they were adolescents [18]. However, in our center, all youth with type 1 diabetes are routinely recommended for complications testing at age 11 years with diabetes duration of 2–5 years. Moreover, our mortality rates are comparable with population-based studies. Data obtained from the National Death Index were used to determine the cause of death. In Australia, there is a robust system of coroner investigation and reporting to determine cause of death; however, we acknowledge that the deaths attributed to diabetes and other causes were not independently adjudicated. Another limitation is the small sample size; however, an Australian National Diabetes Database (ADDN) [39] has since been established which will contribute to future understanding in this area.

In conclusion, we have demonstrated that mortality is higher in young adults with childhood onset type 1 diabetes, particularly in females, and is associated with elevated AER, systolic blood pressure, and subclinical autonomic dysfunction. These findings may assist in early targeted treatment during adolescence in those at significant increased mortality risk. Several deaths in the cohort may not have been directly caused by diabetes but reflect similar findings in other studies of excess mortality due to accidents and other unnatural causes.

## Figures and Tables

**Table 1:** Characteristics at final visit for cohort, alive, and deceased.

	All	Alive	Deceased	*p* ^^^
*n*	409	395	14	—
Male	196 (48)	188 (48)	8 (57)	0.48
Age (years)	17.4 (16.0–18.9)	17.4 (16.0–18.9)	17.5 (16.4–18.7)	0.85
Duration (years)	9.3 (5.9–12.3)	9.3 (6.0–12.3)	6.9 (5.3–12.1)	0.34
HbA1c	8.8 (7.8–10.0)	8.8 (7.7–10.0)	8.7 (7.9–9.4)	0.76
SBP-SDS	0.43 (0.0–0.92)	0.46 (0.0–0.9)	0.01 (−0.8–0.5)	0.07
DBP-SDS	0.77 (0.33–1.4)	0.77 (0.34–1.4)	0.6 (0.1–1.2)	0.32
BMI-SDS	0.59 (0.1–1.0)	0.61 (0.1–1.1)	0.4 (0.1–0.9)	0.53
Retinopathy	176/388 (45)	173/377 (46)	3/11 (27)	0.22
Elevated AER	115/291 (40)	18/375 (5)	5/10 (50)	0.50
Microalbuminuria	23/277 (8)	23/268 (9)	0/9	0.36
Peripheral neuropathy	62/393 (16)	58/380 (15)	4/13 (31)	0.13
Cardiac autonomic neuropathy	79/391 (20)	73/378 (19)	6/13 (46)	0.02
Abnormal pupillary response	70/232 (30)	67/224 (30)	3/8 (38)	0.65
Resting pupillary diameter < 4.77 mm	22/232 (10)	20/224 (9)	2/8 (25)	0.13

Data are *n* (%) or median (IQR). ^#^Urine sample not available for four of deceased group at last visit. ^^^Comparing deceased vs. alive.

**Table 2:** Predictors of mortality in young adults with type 1 diabetes.^*∗*^

Univariate	Odds ratio (95% CI)	*p*	Multivariable	Odds ratio (95% CI)	*p*
Systolic blood pressure SDS	1.49 (1.12–1.98)	0.005	Systolic blood pressure SDS	2.17 (1.26 – 3.74)	0.005
Cardiac autonomic nerve dysfunction	4.87 (1.78–13.31)	0.002	Cardiac autonomic neuropathy	4.65 (1.03 – 21.0)	0.045
Pupillary abnormality	2.54 (0.95–6.76)	0.063	Pupillary abnormality	4.27 (1.20 – 15.22)	0.023
Elevated AER	3.83 (1.45–10.10)	0.007	Elevated AER	4.54 (1.23 – 16.80)	0.030
Peripheral neuropathy	2.15 (1.00–4.59)	0.049	—	—	—
HbA1c	0.81 (0.58–1.12)	0.202	—	—	—
Retinopathy	0.88 (0.35–2.23)	0.785	—	—	—
BMI SDS	1.07 (0.94–1.22)	0.291	—	—	—
Diastolic blood pressure SDS	1.03 (0.93–1.13)	0.591	—	—	—
Albuminuria	1.02 (0.65–1.59)	0.941	—	—	—
Severe hypoglycaemia	0.87 (0.64–1.17)	0.371	—	—	—

^*∗*^Analyses performed using GEE.

## Data Availability

The raw data supporting the conclusions of this article will be made freely available by the authors, with the exception of mortality data which will require the additional approval of the Australian Institute of Health and Welfare.
